# FRAILTY

**DOI:** 10.1093/geroni/igz038.2128

**Published:** 2019-11-08

**Authors:** Odessa Addison

**Affiliations:** Baltimore GRECC VAMHCS, Baltimore, Maryland, United States

## Abstract

The ability to safely maintain mobility function with aging is critical as immobility and falls are among the top reasons for long-term care admissions. One potential cause for these functional deficits are muscle composition changes resulting in reductions in muscle mass, strength and power, ultimately contributing to the development of frailty. While the majority of work examining muscle composition and mobility changes with aging have focused on the quadriceps and ankle plantarflexor/dorsiflexor muscles, accumulating evidence suggests that deficits involving the proximal hip muscles may be particularly harmful to balance and mobility functions leading to falls, hip fractures, and frailty. We will discuss muscle changes that occur with aging and frailty, the implications on mobility, and the effects of potential exercise interventions on muscle structure and function as well as their ability to improve functional mobility.

